# The *Aspergillus niger* Major Allergen (Asp n 3) DNA-Specific Sequence Is a Reliable Marker to Identify Early Fungal Contamination and Postharvest Damage in *Mangifera indica* Fruit

**DOI:** 10.3389/fmicb.2021.663323

**Published:** 2021-06-28

**Authors:** Jorge Martínez, Ander Nevado, Ester Suñén, Marta Gabriel, Ainara Vélez-del-Burgo, Patricia Sánchez, Idoia Postigo

**Affiliations:** ^1^Department of Immunology, Microbiology and Parasitology, Faculty of Pharmacy and Laboratory of Parasitology and Allergy, Lascaray Research Centre, University of the Basque Country, Vitoria-Gasteiz, Spain; ^2^INEGI, Institute of Science and Innovation in Mechanical and Industrial Engineering, Porto, Portugal

**Keywords:** food spoilage, *Aspergillus*, Asp n 3, DNA-marker, fungal food pathogen

## Abstract

The aim of this work was to study the value of the main allergen Asp n 3 of *Aspergillus niger* as a molecular marker of allergenicity and pathogenicity with the potential to be used in the identification of *A. niger* as a contaminant and cause of spoilage of *Mangifera indica.* Real-time polymerase chain reaction (RT-PCR) was used for the amplification of Asp n 3 gene. Two pairs of primers were designed: one for the amplification of the entire sequence and another one for the amplification of the most conserved region of this peroxisomal protein. The presence of *A. niger* was demonstrated by the early detection of the allergenic protein Asp n 3 coding gene, which could be considered a species-specific marker. The use of primers designed based on the conserved region of the Asp n 3 encoding gene allowed us to identify the presence of the closely related fungal species *Aspergillus fumigatus* by detecting Asp n 3 homologous protein, which can be cross-reactive. The use of conserved segments of the Asp n 3 gene or its entire sequence allows us to detect phylogenetically closely related species within the Aspergilaceae family or to identify species-specific contaminating fungi.

## Introduction

Several species belonging to the fungal genus *Aspergillus* are well known as plant pathogens that may cause damage in plants as well as spoil postharvest fruit, vegetables, and cereals, resulting in serious agriculture and economic losses (Perrone et al., 2007; Samson et al., 2010; Mahfooz et al., 2017). Although the main substrate of black aspergilli (*Aspergillus* section Nigri) is soil, these fungi are among the most common fungi causing food spoilage and biodeterioration of other materials. Several authors report that the *Aspergillus niger* species complex can be responsible for the postharvest decay of different fresh fruits and some vegetables (Gautam et al., 2011; Sharma, 2012). It has been demonstrated that *Aspergillus* spores are one of the most frequently identified fungal conidia in the atmosphere (Kasprzyk, 2008; Dijksterhuis, 2019). Its extensive ubiquity in nature probably justifies why this species is one of the most common contaminating fungal genera recorded in various agricultural commodities, such as fruit, nuts, beans, cereals, and vegetables (Samson et al., 2010). In particular, *A. niger* can produce rotting of numerous fruits and vegetables, causing a substantial economic loss. Onions, mangoes, grapes, or tomatoes are the most cited fruits to which *A. niger* can be the major cause of rot phenotype plant disease (Gautam et al., 2011). On the other hand, selected *A. niger* strains are also used in agriculture as promising beneficial microbes (Lubna et al., 2018; Pertile et al., 2021).

*Aspergillus niger* is described as a producer of a wide variety of toxic metabolites, including ochratoxins, a group of secondary metabolites that are classified as possible human carcinogens (Blumenthal, 2004; Tian et al., 2015). In addition to producing toxins, *Aspergillus* species are an important cause of allergy as a relevant source of allergen proteins that are involved in the development of allergic symptoms, particularly in atopic individuals (Simon-Nobbe et al., 2008; Gabriel et al., 2016b).

Among the several allergens described in this mold, the major *A. niger* allergen, Asp n 3, which is homologous to Asp f 3 of *Aspergillus fumigatus* (peroxisomal protein), has been demonstrated to be a clinically relevant component because 72% of the *Aspergillus* sensitized population shows reactive IgE specific to this allergen (Hemmann et al., 1997). This component is also a key marker to use in the differential diagnosis of some different types of aspergillosis (Crameri et al., 1998).

The fungal contamination of fruit with toxins or allergens can lead to a significant health risk to humans. Fruit colonization with these fungi might be used as a direct indicator of fruit or plant damage, food quality, and safety for human consumption (Battilani et al., 2008). Thus, to minimize the fungal diseases associated with both economic losses and health risk, it is essential to detect and identify pathogens at an early stage of the infection process (Amalaradjou and Venkitanarayanan, 2008). In fact, the early and accurate detection and identification of pathogens will allow the control of the spread of fungal infections as well as the implementation of disease management strategies.

The traditional identification of fungi by means of methodologies based on morphological characteristics has a low degree of sensitivity and requires considerable expertise. To address these shortcomings, in recent years, molecular methods such as polymerase chain reaction (PCR) have been considered suitable alternatives for the rapid and early diagnosis of fungal contamination (Gabriel et al., 2015, 2017).

With this in mind, the objective of this work was to study the value of the main allergen Asp n 3 of *A. niger* as a molecular marker of allergenicity and pathogenicity, with the potential to be used in the identification of *A. niger* as a contaminant and cause of spoilage of *Mangifera indica*. Its ability to identify closely related species of the genus *Aspergillus* was also analyzed. The specific detection of Asp n 3 was evaluated in an experimental infection of *M. indica* fruit with *A. niger*, according to Gabriel et al. (2017), by a recently developed PCR method based on the Alt a 1 coding gene for *Alternaria alternata*.

## Materials and Methods

### Fungal Strains and Culture Conditions

The *A. niger* strain CECT 20156 Spanish Type Culture Collection (CECT, University of Valencia, Spain) and the *A. fumigatus* AF54 strain (Laboratory of Parasitology and Allergy, Lascaray Research Centre, University of the Basque Country, Vitoria-Gasteiz, Spain) were used in this study.

*Aspergillus niger* was grown on malt extract agar plates for 4 days at 25°C. The spores were collected in 0.2% agar and 0.05% Tween 80 solution. Then, the spore suspension was diluted 1:100, counted in a Neubauer chamber, and adjusted to a final concentration of 1 × 10^6^ spores/ml with sterile phosphate-buffered saline (PBS) and 0.05 Tween 80 (Gabriel et al., 2017).

### Fruit Inoculation and Incubation

Twenty fresh mangoes were sterilized by immersion in a solution of 1% (v/v) sodium hypochlorite for 1 h at room temperature. Later, they were rinsed out with sterile distilled water and dried out. Sixteen fruits were inoculated with 100 μl of the spore suspension and four mangoes with 100 μl of sterile distilled water as the negative control under sterile conditions. Inoculation was performed by the injection of the spore suspension at 3 mm deep under the fruit skin. Then, the mangoes were placed in polypropylene bags which were closed and incubated at 25°C for 2, 4, 8, and 14 days.

The infection areas in the fruit were measured daily. Contours of the infected zones were drawn into transparent adhesive tape, and the areas were measured by AutoCAD. The negative controls were also checked daily to discard any spontaneous fungal contamination growth.

### Sample Collection and Processing

Four samples from four different fruits corresponding to 2, 4, 8, and 14 days of incubation were analyzed. Samples of 2 cm^2^ × 1 cm deep were obtained from the fruit area where the inoculation was performed. Half of the sample was frozen at −80°C for further PCR analysis, and the other half was incubated in Czapek Dox agar medium at 25°C until visible growth. The negative controls were subjected to the same procedure (Gabriel et al., 2017). *A. niger* positive control and *A. fumigatus* specificity control for PCR were grown on Czapek Dox broth.

### RNA Extraction

The samples were ground in liquid nitrogen with a mortar and pestle. Previously, the material was treated with 70% ethanol, and RNAses were removed by RNAse Zap solution (Thermo Fisher Scientific, Waltham, MA, United States). Then, 4.5 μl of 2-mercaptoethanol was added to 100 mg of the pulverized sample, and Qiagen RNeasy Plant Mini Kit (Qiagen, Hilden, Germany) was used to extract the total RNA according to the instructions provided by the manufacturer (Gabriel et al., 2017).

### cDNA Synthesis, Primer Design, and PCR Amplification

**FIGURE 1:**
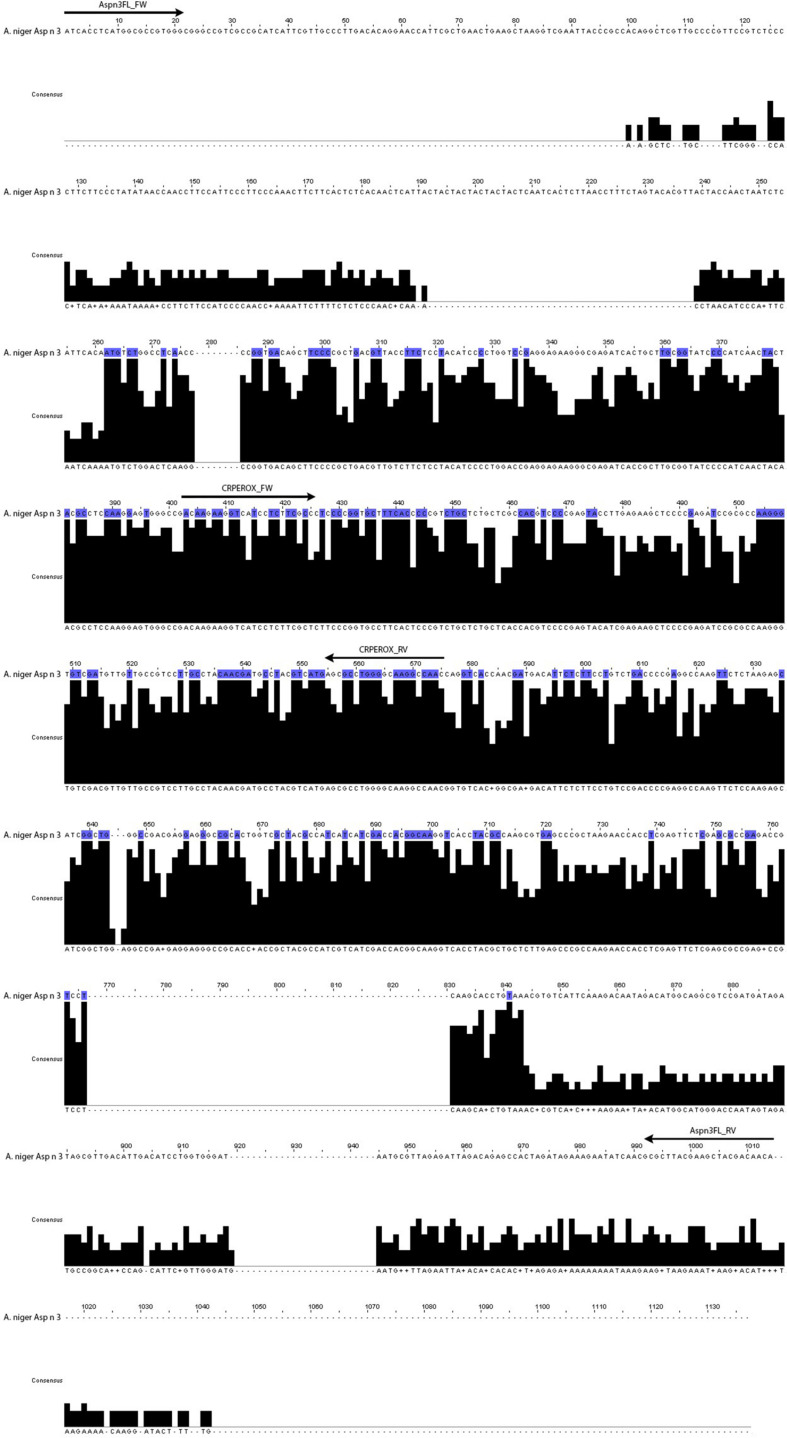
Complete sequence of the gene that codifies for the allergen Asp n 3 compared to the consensus sequence of the fungal species considered in the design (*A. niger*, *A. fumigatus*, *A. terreus*, *A. clavatus*, *A. nidulans*, *A. flavus*, *A. oryzae*, *Penicillium citrium*, *P. chrysogenum*, *Trichophyton rubrum*, and *Neurospora crassa*). The arrows indicate the position of the two pairs of primers corresponding to complete cDNA gene and conserved region.

The reverse transcription reactions were carried out with RevertAid First Strand cDNA Synthesis Kit (Fermentas, Sankt Leon-Rot, Germany). Two pairs of primers were manually designed by comparison of the complete sequence that encodes Asp n 3 with the consensus sequences of the related fungal species considered in the design (Figure 1). The GenBank accession numbers of fungal species used for the multiple alignment analysis by the ClustalW program are shown in Table 1.

**TABLE 1:** GenBank accession numbers of the sequences used in multiple alignment analysis.

Species name	GenBank accession
*Aspergillus niger* CBS 513.88	XM 001395871.2
*Aspergillus terreus* NIH2624	XM 001209908.1
*Aspergillus fumigatus* ATCC 42202	U58050.1
*Aspergillus clavatus* NRRL 1	XM 001270302.1
*Aspergillus nidulans* FGSC A4	XM 676869.1
*Aspergillus flavus* NRRL3357	XM 002380885.1
*Aspergillus oryzae* RIB40	AB226160.1
*Penicillium chrysogenum* Wisconsin 54-1255	XM 002566221.1
*Penicillium citrium* 52-2	AF144753.1
*Trichophyton rubrum* CBS 118892	XM 003238084.1
*Neurospora crassa* OR74A	XM 959107.3

The primers named Aspn3FL_FW (ATCACCTCATG GCGCCGTGGG) and Aspn3FL_RV (TGTTGTCGTA GCTTCGTAAGCGC) were designed to amplify the complete Asp n 3 gene of *A. niger*, including the non-conserved regions of the gene.

The primers named CRPEROX_FW [CAAGAAGGT(C/T) (A/G)T(C/T)CTC(T/G)TCGCC] and CRPEROX_RV (TGTT GTCGTAGCTTCGTAAGCGC) were designed on the basis of the highly conserved internal nucleotide region of the Asp n 3 gene sequence of *A. niger* and its homologs in the other *Aspergillus* species (Gabriel et al., 2017).

Polymerase chain reactions were carried out with a FastStart Taq DNA polymerase PCR kit (Roche, Mannheim, Germany), and a PCR-grade deoxynucleoside triphosphate set (Roche, Mannheim, Germany) was used, following the manufacturer’s instructions. Separate PCRs were performed for each pair of primers. The RT-PCR mixture contained 38.6 μl of water, 5 μl of buffer 10× with 20 mM MgCl_2_, 2 μl of each primer, 1 μl of dNTP mix (10 mM), 0.4 μl of FastStart Taq DNA polymerase, and 1 μl of cDNA. The program set for the Aspn3_FL primers started at 95°C for 4 min and then proceeded with 35 cycles consisting of the first 30 s at 95°C, 30 s at 62°C, and 75 s at 72°C. The program set for the CRPEROX primers started with natural denaturation at 95°C for 4 min followed by 35 amplification cycles consisting of 30 s at 95°C, 30 s at 53°C, and 45 s at 72°C. After that, there was a final extension phase at 72°C for 7 min in both cases. The RT-PCR detection limit was 1 × 10^4^ spores of *Aspergillus*.

Sequencing reactions were performed using the BigDye Terminator, v.3, sequencing kit (Applied Biosystems) according to the manufacturer’s specifications, and the reactions were run in an ABI 3130XL sequencer (Servei de Genòmica, Universitat Autònoma de Barcelona). The amplicon sequence identity was confirmed with NCBI BLAST.

### Alignment of Homologs to Asp n 3 Gene Sequences and Phylogenetic Tree

A BLAST sequence alignment of the Asp n 3 gene with the genes encoding peroxisomal homologous proteins from all expressing species included in the database was carried out. A phylogenetic tree of the nearest neighborhood of representatives constructed based on the nucleotide sequences of Asp n 3 homologs (peroxisomal protein) was constructed using Molecular Evolutionary Genetics Analysis (Tamura et al., 2007).

## Results

Inoculation with *A. niger* resulted in a depressed, circular, brown, soft area surrounding the point of insertion of the pathogen. That area grew, softening the tissues of the mango and cracking the skin. The fungus was able to form spores in the areas where the flesh of the fruit was exposed. No new point of infection was observed during the experiment.

**FIGURE 2:**
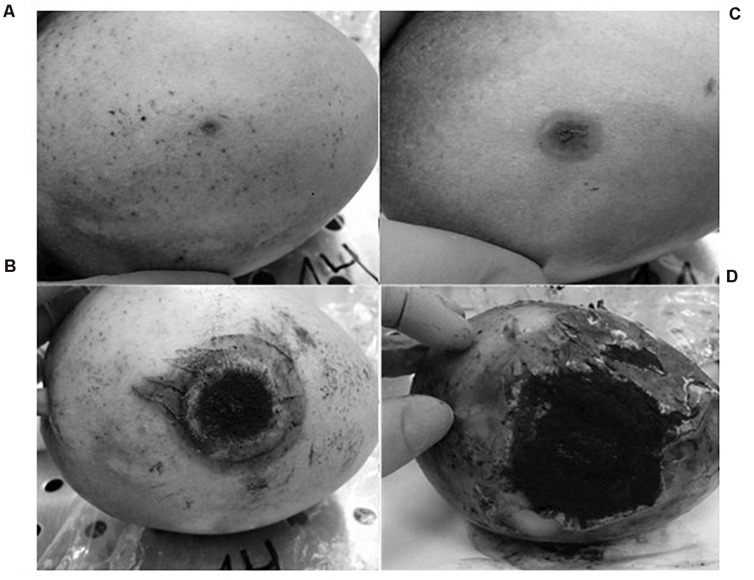
Evolution of the injuries developed by *A. niger* in mango fruit: **(A)** 2 days post-infection (p.i.), **(B)** 4 days (p.i.), **(C)** 8 days (p.i.), and **(D)** 14 days (p.i.).

**FIGURE 3:**
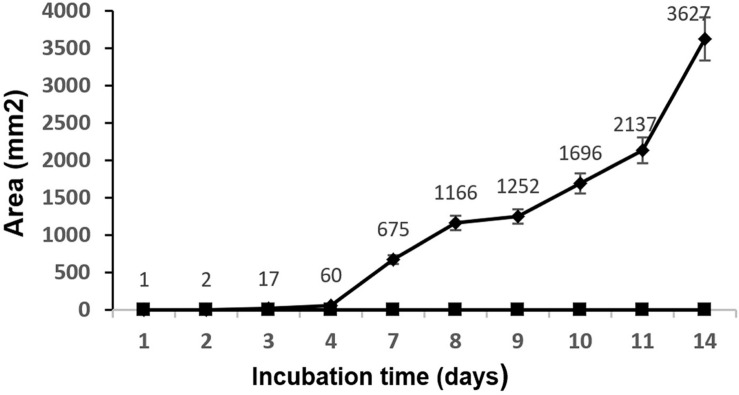
Mean values (mm^2^) and standard deviation of the injured area by *A. niger* in mango fruit. ◆, samples infected with *A. niger* spores; ■, samples inoculated with sterile phosphate-buffered saline.

At 2 days post-inoculation, the lesions provoked by *A. niger* were evident in 100% of the inoculated mangoes. These lesions grew until they covered large areas of the fruit surface (Figure 2). The injuries caused by *A. niger* inoculation grew exponentially, showing lesions 0.8, 3.4, and 6 cm in diameter after 4, 8, and 14 days of incubation, respectively (Figure 3).

The cultures of samples of infected fruit in mycological media were positive for *A. niger* after 3–4 days of incubation. The samples from fruit inoculated with sterile PBS did not show fungal growth.

**FIGURE 4:**
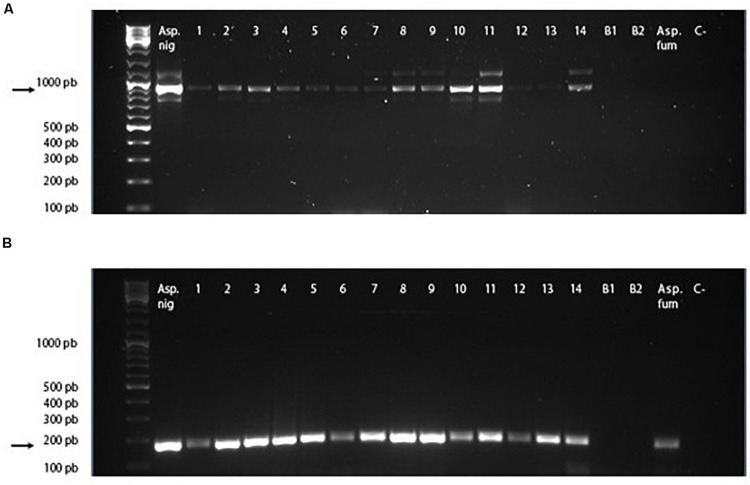
cDNA PCR-amplified regions of Asp n gene. **(A)** Full cDNA gene amplicon using primers *Aspn3_FW* and *Aspn3_RV*. **(B)** Conserved cDNA gene region amplified using primers *CRPEROX_FW* and *CRPEROX_FW*. Asp. Nig, *Aspergillus niger* (positive control). Lines 1–14: samples of mangoes inoculated with spores of *A. niger*. B1 and B2: samples of mangoes inoculated with phosphate-buffered saline. Asp. fum, *Aspergillus fumigatus*. C- :PCR negative control.

The PCR amplicons of the 913-bp complete gene and the 171-bp amplified fragment corresponding to the conserved region of the gene are shown in Figure 4. In 100% of the samples of mangoes infected with *A. niger*, the complete gene and the conserved gene region were amplified. In the sample infected by *A. fumigatus*, only the conserved region of the Asp n 3 gene was amplified, but not the complete region. There was no amplification in the mangoes used as the negative control.

These results confirmed the specificity of the primers designed for the amplification of the Asp n 3 gene for detecting *A. niger* as well as the capability of detecting Asp n 3 cross-reactive proteins using the CRPEROX_FW/CRPEROX_RV primers. This technique allows detection of the presence of the allergen Asp n 3 and its homologs in infected mangoes from the early stages of infection (2 days after inoculation). It is known that the presence of inhibitory substances in food samples may often compromise DNA amplification and lead to the failure of PCR. To prevent false-negative results, positive controls using cDNA obtained from the mycelia of an *A. niger* reference strain (CECT 20156 Spanish Type Culture Collection University of Valencia, Spain) were also included. A sequence analysis of the amplicons allowed the confirmation of the identity of all DNA fragments as Asp n 3. No amplification products were detected in the PCRs using cDNA obtained from PBS-inoculated fruits (Figure 4, B1 and B2 lines).

The presence of *A. niger* was demonstrated by the early detection of the allergenic protein Asp n 3 coding gene, which could be considered a species-specific marker. The use of primers designed based on the conserved region of the Asp n 3 encoding gene allowed us to identify the presence of the closely related fungal species *A. fumigatus* by detecting Asp n 3 homologous protein, which can be cross-reactive. The detection of well-characterized fungal allergens could be considered a tool for identifying fruit rot by *Aspergillus* and to identify fruit contaminated by *Aspergillus* as an allergenic source.

**FIGURE 5:**
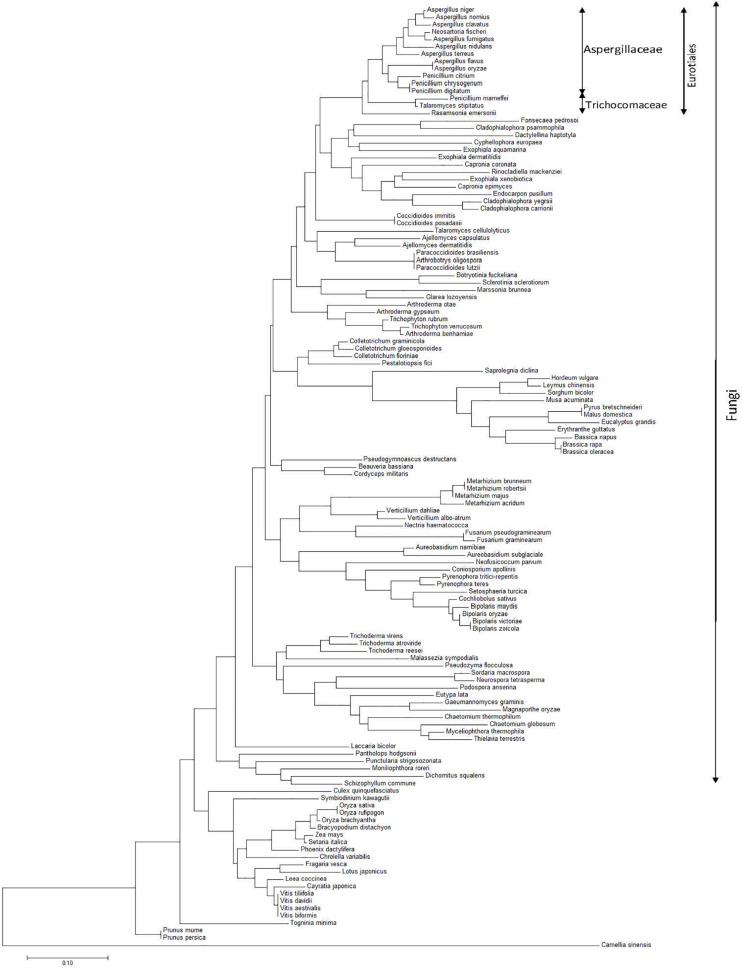
Phylogenetic tree of the nearest neighborhood of representatives constructed on the basis of the nucleotide sequences of Asp n 3 homologs (peroxyredoxin proteins) encoding genes belonging to species from different kingdoms, with special accent on the fungi kingdom.

A phylogenetic tree (based on the nearest-neighbor method) of representatives constructed based on the nucleotide sequences of Asp n 3 homologs (peroxisomal protein) is shown in Figure 5. The sequence alignment of the Asp n 3 homologs by BLAST, belonging to the different species considered to construct the phylogenetic tree, *A. niger*, *Aspergillus terreus*, *Neosartorya fischeri*, *A. fumigatus*, *Aspergillus clavatus*, and *Aspergillus nidulans*, showed more than 90% identity; *Aspergillus flavus* and *Aspergillus orizae* revealed 86% identity; *Penicillium* species showed 81–84% identity; and *Talaromyces* and *Ramsonia* species showed 75–80% identity.

## Discussion

Species from the genus *Aspergillu*s are ubiquitous members of Ascomycotina, which are present in numerous and different habitats (Raper and Fennell, 1965; Christensen and Tuthill, 1986; Bennett, 2010).

*Aspergillus niger* is commonly isolated worldwide from soil, plant debris, air, and indoor environments, and it is considered a soil saprobe and a non-ligninolytic fungus able to produce numerous exoenzymes and organic acids involved in the decomposition cycle of organic matter. These features justify the capability of this fungus to cause the decay of fruits, vegetables, nuts, cereals, wood, or herbs. *A. niger* has also been described as a pathogen for humans and animals as an opportunistic parasite that causes allergic disorders or mycotoxin production (Gautam et al., 2011).

As a plant-pathogenic fungus, *A. niger* has been isolated from a variety of substrates, but its most prominent role is in the rotting or spoilage of numerous fruits, vegetables, and other food products, with relevant economic loss (Sharma, 2012).

The contamination of mango fruit by *A. niger* results in the qualitative deterioration of the product, showing a depressed, circular, brown, soft area surrounding the point where the pathogen starts the colonization (Palejwala et al., 1987; Al-Hindi et al., 2011).

The pathogenicity assay results obtained here showed that the mango fruit is very susceptible to contamination with *A. niger*, and the type of injuries caused by fungal colonization is compatible with that described by other authors (Kim et al., 2020; Shen et al., 2020). These results confirm the validity and applicability of this method for the study of the spoilage of mangoes by *A. niger*.

Because *A. niger* is a well-recognized producer of harmful toxins and significant allergenic proteins, exposure to *A. niger*-contaminated foodstuffs is likely to represent an inherent risk for human health (Simon-Nobbe et al., 2008; Gabriel et al., 2016a). This issue has been underestimated, and studies performed on it are very scarce (Simon-Nobbe et al., 2008; Gabriel et al., 2017). The precise and early detection of *A. niger* contamination would be a relevant added value to establish and prevent health risks and economic losses related to fungal colonization of foods such as fruits and vegetables.

The development of DNA technology has provided effective methods for microbial analysis. In recent decades, genetic studies have provided different nucleic acid markers for microbial identification. These markers are extensively used in PCR or chip-based detection. Based on 16S rDNA and internal transcribed spacer (ITS) sequencing, metagenomic approaches are now emerging technologies for analyzing the entire microbial community in a complex food and vegetable matrix (Shen et al., 2020). Recently, a multiplex RTPCR has been developed, which is able to identify common *Aspergillus* sections quantitatively and detect the presence of azole-resistant strains, allowing the successful detection of early stage fungal colonies within a day of incubation (Kim et al., 2020).

In recent years, several fungal allergens (Alt a 1, Alt a 6, Asp f 1, Asp f 3, Asp n 1, Asp n 3, and others) have been presented as valuable molecular markers of allergenicity and pathogenicity (Matricardi et al., 2016). Accordingly, it is expected that the use of approaches targeting the Asp n 3 gene and/or protein to unequivocally identify and detect the pathogen *A. niger* will yield privileged and powerful information regarding the quality and biosecurity control of foodstuffs (Gabriel et al., 2016b, 2017).

Previously, our research group developed a PCR-based method targeting the gene coding for *A. alternata* major allergen Alt a 1 using species-specific primer sets, and these tools were used to successfully detect the infection of citrus fruit by *A. alternata* early (Gabriel et al., 2017). With this in mind and applying the same concept to the *A. niger* major allergen Asp n 3, further work was conducted to take advantage of the potential of this early detection method to reduce and control postharvest losses during the storage and transportation of fresh mango fruits.

The PCR based on specific Asp n 3 DNA expression gene sequences was able to detect the contamination of mango fruit from the first week of fungal colonization. The amplicons of the complete gene and the amplified fragment corresponding to the conserved region of the gene revealed positive results in 100% of the samples of mangoes infected with *A. niger.* As expected, there was a positive result in the *A. fumigatus* sample for the primers that amplify the conserved region, but not for the primers that amplify the complete Asp n 3 gene.

These results confirmed the specificity of the primers designed for the amplification of the Asp n 3 gene for detecting *A. niger* as well as the capability of detecting proteins susceptible to present cross-reactivity with Asp n 3 using the primers designed to amplify the conserved gene region. This technique allows the detection of the presence of the allergen Asp n 3 and its homologs in infected mangoes from the early stages of the infection (2 days after inoculation). These results are similar to those found by us using the model of *A. alternata* infection in citrus fruit (Gabriel et al., 2017). These results shed light on ways to improve food and vegetable disease prevention, quality, and environmental control.

In the previous study, the gene encoding Alt a 1 defined a protein family associated only with the Pleosporaceae family of fungi (Chruszcz et al., 2012; Gabriel et al., 2017). In contrast, in this study, the peroxiredoxin Asp n 3 belongs to a large protein superfamily associated with numerous species from different kingdoms (Rhee, 2016; Harper et al., 2017). Thus, it could be expected that the specificity for the model studied in this work could be lower than that found previously for the Alt a 1 gene in the infection of citrus fruit with *A. alternata* (Sáenz-de-Santamaría et al., 2006; Gabriel et al., 2015, 2017).

Classically, the macro- and micromorphology of fungi have been the reference methodology for their identification (Visagie et al., 2014). Since the 1990s, DNA sequencing has become one of the most powerful tools for taxonomists because of creating new concepts for identifying the species and defining new tools to establish relationships among them, bringing the opportunity for sequence-based identification (Blaxter, 2003).

During the last decade, ITS rDNA was accepted as the official barcode for fungal identification. Since then, the ITS has been the most widely sequenced marker for fungi, and universal primers for this sequence are available (Schoch et al., 2012). After the introduction of ITS as the most relevant reference marker for taxonomy and phylogenetic relationship studies, in practice, several secondary markers have been analyzed for reliable species identification (Fitzpatrick et al., 2006; Houbraken et al., 2012; Samson et al., 2014; Visagie et al., 2014; Tsang et al., 2018). In *A. niger*, some genes, such as the collagen-like gene, beta-tubulin gene, and calmodulin gene, have been described as secondary markers (Bennett, 2010; Shen et al., 2020).

Peroxiredoxins make up a wide family of proteins associated with several biological functions, including fungal pathogenicity toward their host plants and animals (Chen et al., 2016; Rhee, 2016). The *Aspergillus* group 3 main allergens (Asp f 3, Asp fl 3, Asp n 3, Asp o 3, Asp t 3, Asp ni 3, and Neo fi 3) are peroxisomal proteins. Thus, it would be very interesting to use the peroxiredoxin Asp n 3 as a secondary marker in the identification of the species of this genus and to study the phylogenetic relationships among species (Gautam et al., 2011; Heller and Tudzynski, 2011; Sharma, 2012).

The results presented in this work demonstrate that the Asp n 3 peroxiredoxin gene is an effective tool to identify *A. niger* and establish phylogenetic differences with other species from different taxa. The Asp n 3 peroxiredoxin gene was also demonstrated to be an effective tool to identify pathogenic *Aspergillus* of plant-based foods as well as to identify sources of fungal allergenic sources growing in plant foods.

Fungi from *Aspergillaceae* play diverse and significant roles in biotechnology and human, animal, and plant pathology. Several genomes from *Aspergillaceae* have been studied, including the two iconic genera *Aspergillus* and *Penicillium*. However, their analysis does not fully reflect the diversity of the family. Understanding the evolution of technologically and medically significant fungi through their intrinsic features requires a robust phylogeny (Steenwyk et al., 2019). Other authors suggest that the conflicting results in the fungal classification are likely associated with various processes, such as incomplete lineage sorting, hybridization, or introgression as well as with analytical issues associated with poor taxon sampling (Yilmaz et al., 2014).

DNA markers such as ITS regions or conserved genes such as calmodulin, tubulin, or RNA polymerase are used to conduct molecular phylogenetic studies on *Aspergillus* species. Despite the fact that phylogenetic trees based on single genes are prone to errors, such analyses are robust and have contributed to improving taxonomic systems (Bennett, 2010).

The alignment of genes coding for peroxiredoxin proteins (homologous to Asp n 3) allowed the construction of an Asp n 3-based neighbor-joining phylogenetic tree of species expressing this family of proteins. A monophyletic clade containing species belonging to Eurotiales can be observed. In turn, this group is divided into two differentiate clades, each one containing species belonging to Aspergillaceae and Trichomonaceae.

In agreement with other authors, *Aspergillus* forms a monophyletic clade closely related to *Penicillium* (Aspergillaceae) (Samson et al., 2014). Within this clade, *A. niger*, *A. nomius*, *A. clavatus*, *N. fisheri*, and *A. fumigatus* form the closest *Aspergillus* species subgroup from the phylogenetic point of view. On the other branch, *A. nidulans*, *A. terreus*, *A. flavus*, *Aspergillus oryzae*, *Penicillium citrinum*, *Penicillium chrysogenum*, and *Penicillium digitatum* complete a second subgroup of species within the Aspergillaceae monophyletic clade closely related to *Penicillium*. Both subgroups matched Aspergillaceae.

*Penicillium* (*Talaromyces*) *marneffei*, *Talaromyces stipitatus*, and *Ramsonia emersonii* form a polyphyletic group matching Trichomonaceae, differentiating from the monophyletic clade containing *Aspergillus* and *Penicillium* species.

Some authors suggest the transference of *Penicillium* subgenus *Biverticillum* to *Talaromyces*, and others separate *Ramsonia* from the other genera of Trichocomaceae using a combination of phenotypic characters, extrolite patterns, ITS, and partial calmodulin and b-tubulin sequences (Houbraken et al., 2012; Yilmaz et al., 2014).

The other monophyletic clade, including the species from *Fonsecaea pedrosoi* to *Cladophialophora carrionii*, is the nearest phylogenetic group to the clade that contains Eurotiales species. This clade matches species belonging to Chaetothyriales. The results of this work suggest that Chaetothyriales is the closest related order to Eurotiales within the class Eurotiomycetes.

A phylogenetic analysis revealed a high level of divergence of Asp n 3 among filamentous fungi. The Asp n 3 peroxiredoxin DNA sequence is a marker able to identify *A. niger* species. The use of a highly conserved internal nucleotide region makes it possible to detect, but not to identify, other *Aspergillu*s species.

The alignment of peroxiredoxin nucleotides showed more than 86% identity among species from the *Aspergillus* genus, 81–84% identity when *Penicillium* species were included, and 76–80% identity when the alignment was extended to *Talaromyces* and *Ramsonia*, taking into account that all these genera belong to Eurotiales.

These results justify the detection of *Aspergillu*s species, but not their identification, when only highly conserved regions are used. Species identification requires tools based on full gene sequences.

Previously, some authors demonstrated that cytochrome B coding genes or small heat shock protein coding genes were useful for the most accurate classification of fungi (Yokoyama et al., 2001; Wu et al., 2016). In our case, we demonstrate that the peroxiredoxin Asp n 3 can be used in the analysis of phylogeny, classification, and identification of aspergilli.

## Conclusion

The Asp n 3 DNA sequence is a reliable tool to detect and identify early fungal contamination by *Aspergillus* and postharvest damage in *M. indica* fruit. The use of conserved segments of the Asp n 3 gene or its entire sequence allows us to detect phylogenetically closely related species within the Aspergilaceae family or to identify species-specific contaminating fungi.

## Data Availability Statement

The original contributions presented in the study are included in the article/supplementary material, further inquiries can be directed to the corresponding author.

## Author Contributions

JM, IP, and ES designed the experiments. AN and MG performed the experiments. AN, AV-d-B, and PS conducted bioinformatics for the amplicon sequencing results and phylogenic studies. JM and IP wrote the manuscript. ES formatted the document. All the authors reviewed and revised the manuscript.

## Conflict of Interest

The authors declare that the research was conducted in the absence of any commercial or financial relationships that could be construed as a potential conflict of interest.
